# Does Thirty-Minute Standardised Training Improve the Inter-Observer Reliability of the Horse Grimace Scale (HGS)? A Case Study

**DOI:** 10.3390/ani10050781

**Published:** 2020-04-30

**Authors:** Francesca Dai, Matthew Leach, Amelia Mari MacRae, Michela Minero, Emanuela Dalla Costa

**Affiliations:** 1 Dipartimento di Medicina Veterinaria, Università degli Studi di Milano, via Celoria 10, 20133 Milano, Italy; francesca.dai@unimi.it (F.D.); michela.minero@unimi.it (M.M.); 2 School of Natural and Environmental Sciences Agriculture, Newcastle University, Agriculture Building, Newcastle Upon Tyne NE1 7RU, UK; matthew.leach@newcastle.ac.uk; 3 Animal Welfare Program, University of British Columbia, 2357 Main Mall, Vancouver, BC V6T 1Z4, Canada; amarimacrae@gmail.com

**Keywords:** HGS, horse, pain assessment, training, welfare assessment

## Abstract

**Simple Summary:**

The recognition of pain in equine practice is highly dependent on the assessors’ reliability in using pain assessment tools. The Horse Grimace Scale (HGS) is one such tool, a facial-expression-based pain coding system able to identify a range of acute painful conditions in horses. This study aimed at evaluating the efficacy of a standardised HGS training program at improving the agreement of assessors without horse experience by comparison with an expert. The results suggest that 30-minute face-to-face training may not be sufficient to allow observers without horse experience to effectively learn about HGS and its consentient facial action units to then be able to effectively apply this scale. The training method applied could represent a starting point for a more comprehensive training program for assessors with no experience.

**Abstract:**

The Horse Grimace Scale (HGS) is a facial-expression-based pain coding system that enables a range of acute painful conditions in horses to be effectively identified. Using valid assessment methods to identify pain in horses is of a clear importance; however, the reliability of the assessment is highly dependent on the assessors’ ability to use it. Training of new assessors plays a critical role in underpinning reliability. The aim of the study was to evaluate whether a 30-minute standardised training program on HGS is effective at improving the agreement between observers with no horse experience and when compared to an HGS expert. Two hundred and six undergraduate students with no horse experience were recruited. Prior to any training, observers were asked to score 10 pictures of horse faces using the six Facial Action Units (FAUs) of the HGS. Then, an HGS expert provided a 30-minute face-to-face training session, including detailed descriptions and example pictures of each FAU. After training, observers scored 10 different pictures. Cohen’s k coefficient was used to determine inter-observer reliability between each observer and the expert; a paired-sample *t*-test was conducted to determine differences in agreement pre- and post-training. Pre-training, Cohen’s k ranged from 0.20 for tension above the eye area to 0.68 for stiffly backwards ears. Post-training, the reliability for stiffly backwards ears and orbital tightening significantly increased, reaching Cohen’s k values of 0.90 and 0.91 respectively (paired-sample *t*-test; *p* < 0.001). The results suggest that this 30-minute face-to-face training session was not sufficient to allow observers without horse experience to effectively apply HGS. However, this standardised training program could represent a starting point for a more comprehensive training program for those without horse experience in order to increase their reliably in applying HGS.

## 1. Introduction

Using valid assessment methods to identify pain in horses as a consequence of husbandry practices or in a clinical setting is of a clear importance [1,2]. However, whatever assessment method is chosen, its reliability (repeatability in time and consistency within and between observers [3]) is highly dependent on the assessors’ ability to use it. Several factors can complicate the recognition of pain in horses. They are a prey species and therefore may hide their pain [4]; moreover, individual temperament has been shown to influence the intensity that pain-related behaviours are exhibited [5]. A training program aiming to improve the accuracy of pain evaluation by new assessors should be developed in order to improve their inter-observer reliability [6,7]. This would guarantee that the use of pain indicators by multiple individuals will provide reliable results, thus more consistently reflecting pain levels observed, and be applicable in daily clinical practice [8,9]. Well-designed training programs are especially important for equine pain assessment, given the diversity observed in the horse industry, in terms of breeds, different housing systems, various disciplines, different professional levels [10] and the variability in background (i.e., experience, knowledge, etc.) of people involved in the sector (e.g., horse caretakers, veterinarians, owners, etc.).

The Horse Grimace Scale (HGS) is a facial-expression-based coding system, which can be used to recognise pain in horses [2,11,12,13]. It includes six Facial Action Units (FAUs): stiffly backwards ears, orbital tightening, tension above the eye area, prominent strained chewing muscles, mouth strained and pronounced chin and strained nostrils. A score of 0 indicates high confidence of the observer that the action unit was absent. A score of 1 indicates either high confidence of a moderate appearance of the action unit or equivocation over its presence or absence. A score of 2 indicates high confidence of a marked appearance of the action unit. Facial expressions are particularly useful in pain assessment, as they cannot be completely suppressed by voluntary control, and importantly this is still evidenced in prey species [14,15]. It has been shown that a short training period for new HGS assessors is sufficient to allow them to reliably apply this method with a good inter-observer reliability [11,13]. However, in the above-mentioned studies, the new HGS assessors involved were experienced veterinarians familiar with normal species-specific behaviours. Untrained assessors with different backgrounds and experience could represent a possible bias in the evaluation of the efficacy of a training program [16]. Therefore, the aim of a successful training program should ensure high reliability irrespective of the different background experience of the observer [17]. No data are currently available regarding how observers without previous experience in either in pain assessment or horse behaviour can learn to apply the HGS reliably by comparison to HGS experts.

The present study aimed to evaluate whether a standardised face-to-face training program that combined theory and practical experience was effective at improving and ensuring the reliability of observers with no horse experience when utilising the HGS, measured in terms of inter-observer reliability.

## 2. Materials and Methods

### 2.1. Ethic Statement

All students were verbally informed about the methods and the objectives of the research and the data collection, and they entered the study on a voluntary basis. At any time, students could withdraw their consent. No sensitive data were collected, and it was not possible to identify the participants from the raw research data.

### 2.2. Students

Undergraduate students (n = 206) from five institutions voluntarily participated in the study (Table 1). Inclusion criteria were that participants had no direct experience with horses and were unfamiliar with the Horse Grimace Scale scoring system.

### 2.3. HGS Standardised Training Program

An HGS expert (an academic scientist renowned internationally for her expertise in horse welfare, who has previously scored over 200 pictures using HGS) provided a 30-minute face-to-face training session. This training included a presentation of the HGS scoring system, detailed descriptions of each Facial Action Unit (FAUs) with example pictures and examples of images that had previously been scored by the HGS expert. The students were encouraged to interact with the trainer, ask questions and actively discuss the method and the scoring of example pictures.

### 2.4. Data Collection

**Figure 1:**
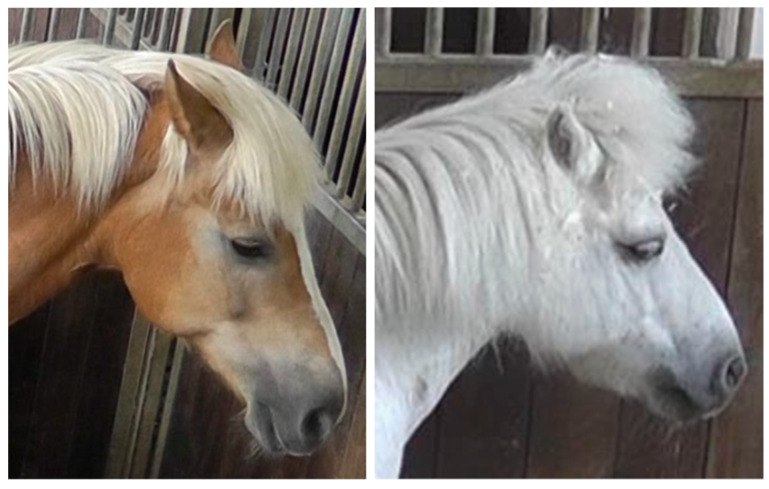
Example of pictures scored by the students.

Twenty previously scored pictures showing a profile view of the head of different breeds and colours of horses were selected (for an example see Figure 1). The pictures provided were collected from horses in pain due to acute laminitis (previously published data on the HGS [11]). High-quality pictures were selected with the aim of showing a wide range of FAU scores (balancing the number of pictures with scores of 0, 1 and 2 for the different FAUs). Pictures were projected on a screen one at a time. Data were collected in two phases: pre- and post-training. In the ‘pre-training’ phase students first received a brief lecture on the definition of pain and its effect on facial expressions in different species (e.g., mice, rats, rabbits) but not horses. They then were asked to score 10 pictures of horse faces. They were not introduced to the HGS in this phase. In the ‘post-training’ phase students received the HGS standardized training outlined in Section 2.2 and then scored a second different set of 10 pictures. All pictures were also scored by an HGS expert (E.D.C.).

### 2.5. Statistical Analysis

The Intraclass Correlation Coefficient (ICC) has been used in other studies to assess the reliability of grimace scales when scored by several observers with similar experience (interchangeable observers). However, the aim of the present study was to compare the HGS scores of an expert to those of observers (non-interchangeable due to the different experience) with no experience with horses. Therefore, Cohen’s kappa coefficient was used to determine inter-observer reliability between each student and an HGS expert. The kappa statistic rages from 0 to 1 and can be interpreted as follows [18]: agreement equivalent to chance (less than 0.10); slight agreement (0.10–0.20); fair agreement (0.21–0.40); moderate agreement (0.41–0.60); substantial agreement (0.61–0.80); near perfect agreement (0.81–0.99); perfect agreement (1). All statistical analyses were conducted using SPSS 25 (SPSS Inc., Chicago, IL, USA). The data were tested for normality and homogeneity of variance using Kolmogorov–Smirnov and Levene tests, respectively. Paired-sample *t*-tests were conducted to determine if there was a significant difference in agreement between the students and the expert from pre- to post-training. Differences were considered to be statistically significant at *p* ≤ 0.05.

## 3. Results and Discussion

**Figure 2:**
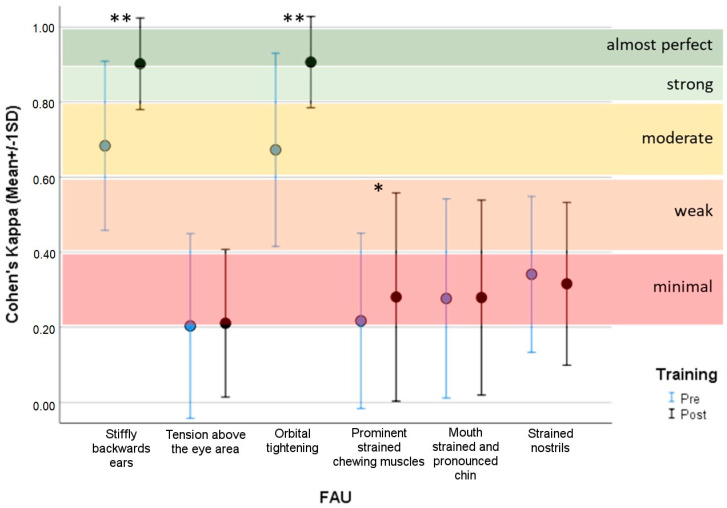
Mean ± SD of Cohen’s Kappa values between observers and a Horse Grimace Scale (HGS) expert pre- and post-training. Paired-sample *t*-test, ** *p* < 0.001 * *p* < 0.05.

The training protocol presented in this paper was previously applied to a smaller number of trainees without horse experience to assess inter-observer reliability [19]. It showed that reliability was excellent before training with an Intraclass Correlation Coefficient of 0.986, and then improved after 30 min of training to 0.992 (both high degrees of reliability). However, this study did not evaluate the agreement between observers with no horse experience with that of an expert, which is critical for determining the efficacy of training naive observers [16,20]. The results of the present study showed a high variability of agreement between naïve observers and the expert for the different facial action units comprising the HGS: ranging from 0.20 for tension above the eye area to 0.68 for stiffly backwards ears (Figure 2). Only stiffly backwards ears (Cohen’s kappa = 0.68) and orbital tightening (Cohen’s kappa = 0.67) reached a substantial agreement before training, while all other FAUs only showed slight agreement or fair agreement. Following training, the agreement for stiffly backwards ears and orbital tightening significantly increased, reaching Cohen’s kappa values of 0.90 and 0.91 respectively, indicating near perfect agreement (paired-sample *t*-test; *p* < 0.001); the agreement for prominent strained chewing muscles significantly increased to 0.28 indicating only a fair agreement (paired-sample *t*-test; *p* < 0.05). For the other FAUs, no significant modification of Cohen’s kappa value was observed from pre- to post-training. Interestingly, stiffly backwards ears and orbital tightening were the same FAUs that showed the highest inter-observer reliability (ICC) in the previous studies that had a smaller number of trainees with and without horse experience [11,19]. A possible explanation for this result is that these two FAUs seem rather easy to assess and robust.

These results indicate that the 30-minute face-to-face standardised training of naïve observers without any horse experience was not sufficient to reach a good agreement with an HGS expert for the majority of the FAUs. Studies of other welfare (e.g., body condition score) or clinical (e.g., skin lesions) indicators have obtained similar results [16,21]. Consequently, the development of more effective training programs for welfare indicators is imperative to ensure welfare is assessed effectively and reliably, and this is particularly important for pain assessment. The training method utilised here obtained a significant improvement in the agreement between naïve observers and the expert for three out of six FAUs. A possible explanation for the lack of change in the remaining FAUs could be image quality, which can be defined as “the weighted combination of all of the visually significant attributes of an image” [22]. High-quality pictures are required to more easily allow observers to identify the characteristics of each FAUs and detect differences between scores effectively; in our study, pictures were obtained from clinical settings with different lighting, sharpness, noise, contrast, artefacts and colour, so individual image quality varied. Due to the clinical setting, it was not always possible to capture each horse from the perfect angle to facilitate the most effective scoring, and this may influence the ability of the naive observers to recognise the different FAUs, in particular the above-eye area, the nostrils and the mouth. Another possible explanation is that the pictures were projected on a screen; this procedure was different from those reported in previous studies [11,13] where the pictures were presented on a monitor with high-quality resolution. In a previous study where the same images were scored by two trained veterinarians, lower ICC scores were recorded for the same FAUs [11], confirming that these FAUs could be more difficult to score. The FAU descriptions used to train the observers were those reported by Dalla Costa and colleagues [13], and so more detailed descriptions maybe needed to better clarify each FAU for a naïve assessor with no horse experience. Considering these results, including videos and live scoring could be a more effective training for improving the reliability of these FAUs. Vasseur and colleagues demonstrated that an in-depth description of each body condition score is needed to obtain a high inter-observer reliability, and that the use of a simple chart was not enough to assure assessor agreement [16]. The same study also highlighted the need for observers to be exposed to “extreme” examples of the scores (e.g., Body Condition Score = 1 and Body Condition Score = 5) to allow the observers to differentiate extreme from normal conditions [16]. In this study, we showed example pictures illustrating the different scores during the training; however, the number of pictures may have been insufficient for the naïve observers to clearly differentiate and memorize the different characteristics of each FAU. Since the goal of our study was to investigate the efficacy of a short face-to-face training, we chose only 30 min. This period may not have been long enough to allow observers without horse experience to effectively internalize the methods and efficiently apply them. In addition, the large number of observers per class did not allow a deep one-to-one exchange between each observer and the trainer. As a consequence, when using facial-expression-based scoring in a clinical situation, training should be planned in order to ensure new assessors’ competency in the field. As it has been demonstrated that the sole use of educational material (images) as a training tool is insufficient [16], mixed methods of training, using both pictures and live animals during the scoring process, may provide better results in term of inter- and/or intra-observer reliability [16,23,24]. Gibbons et al. [23] highlighted that if trainees do not meet a target level of agreement, they should not be used for on-farm data collection, in research or in commercial farm evaluation. More needs to be done to design a training protocol for HGS, which could be applied to prepare new assessors without horse experience to ensure reliable assessment of the HGS and pain.

## 4. Conclusions

Our results suggest that the training program applied could represent a starting point for a more comprehensive training program for observers without horse experience in order to teach them how to reliably apply HGS. However, a dedicated picture collection composed of high-quality and uniform pictures, and a more extensive training program involving a lower number of observers per trainer, may be necessary. Finally, a session in which observers can practice scoring live animals seems fundamental for improving the accuracy of in-field pain evaluation.

## Figures and Tables

**Table 1:** Number of recruited students from each institution.

Course	Institution	n of Students
Second year students in Veterinary Medicine	University of Milan	n = 63
Fourth year students in Veterinary Medicine	University of Teramo	n = 31
Third and fourth year students of Applied Biology	University of British Columbia	n = 28
Third year and MSc students in Animal Science	University of Newcastle	n = 40
Second and third year students in Animal Welfare and Husbandry	University of Milan	n = 44
